# Effects of COVID‐19 Lockdowns on Alcohol Consumption, Hangovers and Smoking Among Young Adults (*n* = 140) in Germany: An On‐Line Study

**DOI:** 10.1002/hup.70000

**Published:** 2025-01-30

**Authors:** Agnese Merlo, Anna H. Koyun, Pauline A. Hendriksen, Johan Garssen, Gillian Bruce, Ann‐Kathrin Stock, Joris C. Verster

**Affiliations:** ^1^ Division of Pharmacology Utrecht Institute for Pharmaceutical Sciences Utrecht University Utrecht The Netherlands; ^2^ Cognitive Neurophysiology Department of Child and Adolescent Psychiatry Faculty of Medicine Dresden Germany; ^3^ Danone Global Research & Innovation Center Utrecht The Netherlands; ^4^ School of Education and Social Sciences University of the West of Scotland Paisley UK; ^5^ Centre for Mental Health and Brain Sciences Swinburne University Melbourne Australia

**Keywords:** alcohol, COVID‐19, hangover, lockdown, smoking, tobacco

## Abstract

**Objective:**

This study investigated the impact of 2019 coronavirus disease (COVID‐19) lockdowns on alcohol consumption and smoking behavior among young adults from Germany.

**Methods:**

An online survey was completed by *N* = 317 young adults living in Germany. Of these, 140 (44.2%) consumed alcohol and were included in the analysis. They reported on alcohol consumption, hangover frequency and severity, and smoking behavior across four time periods: (1) “BP” (the period before the COVID‐19 pandemic), (2) “L1” (the first lockdown; March–May, 2020), (3), NL1 (the no lock‐down period; summer 2020), and (4) L2 (the second lockdown, November 2020 to May 2021).

**Results:**

During L1, a significant decrease was observed in weekly alcohol intake, the number of drinking days, and the number of days where more than eight alcoholic drinks were consumed. Whereas hangover frequency was significantly increased during L1, hangover severity was significantly reduced. During NL1, drinking behaviors returned to BP levels. During L2, the decrease in alcohol consumption was much smaller, and not significantly different from BP. However, compared to BP, during L2 hangover frequency was significantly increased and hangover severity was significantly reduced. With regards to smoking, no significant differences compared to BP were found.

**Conclusions:**

During the COVID‐19 lockdown periods in Germany, a significant decrease in alcohol consumption was observed among this group of young adults. Whereas hangover frequency was significantly increased during the lockdown periods, hangover severity was significantly reduced.

## 1 Introduction

Research investigating the effects of lockdowns and stay‐at‐home directives indicated that these measures can exert a substantial adverse influence on psychological well‐being, potentially leading to escalated substance misuse behaviors, specifically increased alcohol consumption and tobacco smoking (Koopmann et al. 2020; Manthey et al. 2021; Jackson et al. 2022). Thus, following the outbreak of the 2019 coronavirus disease (COVID‐19), the risk of escalated alcohol use amid the pandemic surfaced as a public health issue concern. Moreover, the rise in smoking and drinking of alcohol could directly contribute to the severity of COVID‐19 (Dorjee et al. 2020; Wei et al. 2023), also supported by the link between high alcohol use and the increased risk of acute respiratory distress syndrome (ARDS) (Simou, Leonardi‐Bee, and Britton 2018).

Examination of the literature with regards to the risk of alcohol consumption amid the pandemic yields mixed findings. Overall, the research indicates different alcohol use patterns among the surveyed population with some reporting an increase in alcohol consumption (Panagiotidis et al. 2020; Vanderbruggen et al. 2020), and others a reduction in consumption (Lechner et al. 2020; White et al. 2020; Reyerson et al. 2021). Authors have reported several factors associated to the various drinking patterns. For instance, an increase in alcohol consumption is often attributed to being female, younger age, higher educational level, and experiencing certain psychological states such as higher levels of stress and anxiety (Avery et al. 2020; Grossman, Benjamin‐Neelon, and Sonnenschein 2020; Koopmann et al. 2020; Capasso et al. 2021; Killgore et al. 2021; Bloomfield et al. 2022). On the contrary, being a university student would appear to have a protective effect towards alcohol use, with several studies showing a decrease in alcohol consumption among student populations (Evans et al. 2021; Jaffe et al. 2021; Valente et al. 2021; Schmits and Glowacz 2022). Taken together, these findings emphasize that individuals are not uniformly impacted by lockdowns, and motivations for alcohol consumption and drinking behaviors vary significantly among the population (Vanderbruggen et al. 2020; Alpers et al. 2021; Jacob et al. 2021). In previous studies among students of The Netherlands (Hendriksen et al. 2021; Merlo et al. 2021a) and Argentina (Hendriksen et al. 2022; Karadayian et al. 2023), a significant decrease in alcohol consumption, hangover frequency and severity were observed during lockdown periods compared to before the COVID‐19 pandemic. These studies revealed that both before and during the COVID‐19 pandemic females consume significantly less alcohol than males, and experience less severe hangovers. The Argentinian study further reported that older students (25–35 years old) consumed more alcohol than younger students (18–24 years old). However, the majority of the drinking variables assessed during the lockdown and no lockdown periods did not differently affect males versus females or younger versus older students.

Smoking is widely recognized as a risk factor associated with increased vulnerability and heightened severity of respiratory infections, including COVID‐19 (Bellan et al. 2020; Gupta, Nethan, and Mehrotra 2021). Similar to alcohol use, smoking is a common coping mechanism used to relieve stress and tension, Thus a greater prevalence of these behaviors could occur in relation to encountering elevated levels of stress due to factors such as social isolation, employment uncertainties, financial challenges, caregiving responsibilities, and fear of COVID‐19 (Brooks et al. 2020; Fitzgerald, Nunn, and Isaacs 2020). On the contrary, individuals who smoke for social motives (e.g., at parties only) may be less likely to partake in smoking at home. Stay at home mandates may even prompt smokers to attempt to quit, as potentially linked to the inability to or willingness to avoid smoking indoors (Jackson et al. 2021). Mixed results have however been reported on smoking during the COVID‐19 pandemic. Jackson et al. (2021), for example, reported a significant increase in smoking during the first COVID‐19 lockdown in England, but also an increase in the number of attempts to quit smoking among the younger group of 18–34 years old. A French study also reported an increase in tobacco smoking, specifically among respondents aged 18–34 years (Guignard et al. 2021). In contrast, a study conducted among Argentinian students, showed a significant reduction in the number of cigarettes smoked per day during the lockdown periods compared to before the COVID‐19 pandemic (Karadayian et al. 2023), whilst the number of smoking days per week remained unchanged. Further analysis with respect to sex revealed that among males the number of cigarettes smoked was significantly reduced during the second lockdown period. For females, no changes were observed compared to before the COVID‐19 pandemic. Regarding age, older students (25–35 years old) reported smoking more cigarettes per day than younger students (18–24 years old). The difference in number of cigarettes smoked per day between the age groups was significant for the first lockdown period, as younger students significantly reduced the number of cigarettes smoked (5.0–3.7 cigarettes), whereas older students non‐significantly increased the number of cigarettes smoked per day (7.5–10.4 cigarettes). Smoking behavior during the subsequent no lockdown period did not significantly differ from before the COVID‐19 pandemic. During the second lockdown period, a similar effect on smoking behavior was observed, although the difference between the age groups did not reach statistical significance.

The aim of the current analysis was to further investigate alcohol consumption and smoking behavior during the lockdown periods in Germany, and explore their relationship with age (18–24 years old group vs. 25–35 years old group), sex (males vs. females), and occupation (student vs. employee). To this extent, a survey conducted by our team in The Netherlands (Hendriksen et al. 2021; Merlo et al. 2021a) and Argentina (Hendriksen et al. 2022; Karadayian et al. 2023) was replicated and extended in Germany (Koyun et al. 2022). In Germany, the pandemic was characterized by alternating lockdown and no lockdown periods (Koyun et al. 2022). In this study, we evaluated the periods (1) “BP” (the period before the COVID‐19 pandemic), (2) “L1” (the first lockdown period; March–May, 2020), (3), NL1 (the first no lock‐down period; summer 2020), and (4) L2 (the second lock‐down; November 2020 to May 2021). With regards to alcohol use during lockdown periods, in line with previous studies we conducted in Argentina and the Netherlands, it was hypothesized that alcohol consumption and smoking would be decreased during the COVID‐19 lockdown periods (L1 and L2) and returned to BP levels during the no lockdown period (NL1).

## 2 Methods

An online questionnaire was distributed to German young adults aged 18 to 35, with participants recruited through email and printed flyers. The survey, available in both German and English, was conducted using the LimeSurvey online platform (LimeSurvey GmbH, Hamburg, Germany). Participants had the opportunity to enter a prize draw for a chance to win one of four 25 Euro Amazon gift cards. The study spanned from mid‐November 2021 to the end of March 2022. Informed consent was obtained from all participants. Ethical approval was granted by the Ethics Committee of the Medical Faculty of TU Dresden (Approval code: SR‐EK‐8012020, date of approval: 27 September 2021). A detailed description of the study methodology and the dataset are published elsewhere (Koyun et al. 2022).

For the current analysis, only participants who consume alcohol were included. Demographic data were collected, including age (years), sex (male or female), and occupation (job or student). All assessments were made for the periods BP, L1, NL1, and L2. For alcohol consumption, the average number of alcoholic drinks per week was reported as well as the number of drinking days per week. In addition, the number of drinking days per month on which participants consumed more than eight alcoholic drinks was reported. Participants were informed how to convert the amount of alcohol they consumed into standardized alcoholic drink sizes (1 unit equals 10 g alcohol) (Koyun et al. 2022). For alcohol hangovers, the number of hangovers per month was recorded. The average hangover severity for these occasions was rated on a scale ranging from 0 (absent) to 10 (extreme) (Verster et al. 2020). For smoking behavior, the number of smoking days per week and the number of cigarettes smoked per day were recorded.

Data were analyzed with SPSS (IBM Corp. Released 2013. IBM SPSS Statistics for Windows, Version 29.0. Armonk, NY: IBM Corp.). Mean and standard deviation (SD) were computed for all variables. Within‐subject comparisons of alcohol consumption and smoking (comparing the timepoints with BP data) were conducted with the Related‐Samples Friedman's Two‐Way Analysis of Variance by Ranks test. Pairwise comparisons of assessments with BP were considered significant if *p* < 0.0125, that is, after Bonferroni's correction for multiple comparisons. To account for the relative small sample size, bootstrapping was applied. Paired comparisons with BP were conducted for *B* = 10,000 samples, and the bias‐corrected and accelerated (BCa) 95% bootstrapped confidence interval (CI_B_) was computed (Efron and Tibshirani 1993; Sideridis and Simos 2010). The BCa 95% CI_B_ can range from −1 to +1, with a more narrow BCa 95% CI_B_ implying a greater precision. Differences from BP are considered statistically significant if the BCa 95% CI_B_ does not contain zero.

## 3 Results

*N* = 317 individuals participated in the study. *N* = 140 (44.2%) of them consumed alcohol and were included in the analysis. Their demographics are summarized in Table 1.

**TABLE 1:** Demographics.

Assessed variables	Overall
*N*	140
Male/Female ratio (%)	48 (34.3%)/92 (65.7%)
Age	25.3 (4.0)
Smoking (%)	33 (23.6%)
Ethnicity	
German	107 (76.4%)
Western Europe	8 (5.7%)
North America	3 (2.1%)
Non‐Western	19 (13.6%)
Other	3 (2.1%)
Living situation	
Alone	41 (29.3%)
Together with others	66 (47.1%)
Together with family	33 (23.6%)
SARS‐CoV‐2 status	
Not infected	121 (86.4%)
Infected and hospitalized	8 (5.7%)
Infected, but not sick	11 (7.9%)
Occupation	
Student	94 (67.1%)
Employee	46 (32.9%)

*Note:* Mean and standard deviation (SD, between brackets) or percentages are shown.

### 3.1 Alcohol Consumption

**FIGURE 1:**
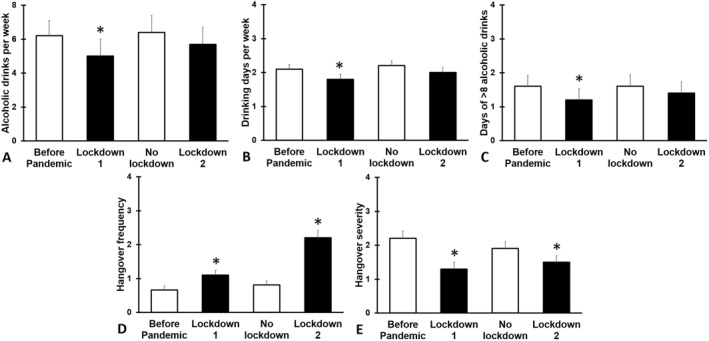
Alcohol consumption and hangovers during the COVID‐19 pandemic. Means and standard errors are shown for the period (1) “Before Pandemic” (the period before the COVID‐19 pandemic), (2) “Lockdown 1” (the first lockdown period, March–May, 2020), (3), “No Lockdown” (the first no lockdown period, Summer 2020), and (4) “Lockdown 2” (the second lockdown, November 2020 to May 2021). Data is shown for (A) number of alcoholic drinks consumed per week, (B) drinking days per week, (C) days per month with more than 8 alcoholic drinks, (D) hangover frequency per month, and (E) average hangover severity. Differences from “before the COVID‐19 pandemic”, that is, if the 95% bootstrap confidence interval (BCa 95% CI_B_) does not include zero, and are indicated by *.

The alcohol consumption data are summarized in Figure 1 and Table 2.

**TABLE 2:** Alcohol consumption.

Overall assessments	Mean (SD)					Pairwise comparisons		
Time period	BP	L1	NL1	L2	Overall	BP versus L1	BP versus NL1	BP versus L2
Number of alcoholic drinks per week	6.2 (10.6)	5.0 (11.5)	6.4 (12.0)	5.7 (11.4)	< 0.001*	0.002*	0.298	0.611
Drinking days per week	2.1 (1.6)	1.8 (1.7)	2.2 (1.7)	2.0 (1.8)	0.002*	0.035*	0.502	0.611
Days with > 8 alcoholic drinks	1.6 (3.9)	1.2 (3.9)	1.6 (4.0)	1.4 (3.9)	< 0.001*	0.008*	0.853	0.179
Hangover frequency per month	1.2 (1.7)	0.7 (1.4)	1.1 (1.7)	0.8 (1.5)	< 0.001*	< 0.001*	0.139	0.007*
Average hangover severity	2.2 (2.6)	1.3 (2.2)	1.9 (2.4)	1.5 (2.3)	< 0.001*	0.002*	0.220	0.022*

*Note:* Mean, standard deviation (SD, between brackets), and *p*‐values are shown. Significant differences between before the COVID‐19 pandemic (BP) and the other time periods are indicated by *. Pairwise comparisons with BP were computed if the main effect was significant (*p* < 0.05). These were considered significant if the 95% bootstrap confidence interval (BCa 95% CI_B_) does not include zero, indicated by *.

Abbreviations: BP = before the COVID‐19 pandemic, COVID‐19 = coronavirus disease 2019, L1 = first lockdown period, L2 = second lockdown period, NL1 = first no lockdown period.

Compared to before the COVID‐19 pandemic, all tested variables for the first lockdown related to alcohol consumption and hangovers, were statistically different from BP. Compared to BP, 49.2% of the sample reported a reduction in weekly alcohol consumption, 35.7% reported no change, and 21.4% reported an increase in weekly alcohol consumption during the first lockdown period. Overall, during L1 a significant reduction was found in weekly alcohol consumption (BCa 95% CI_B_ = −2.13, −0.24), the number of drinking days per week (BCa 95% CI_B_ = −0.45, −0.08), and the number of drinking days on which more than 8 alcoholic drinks were consumed (BCa 95% CI_B_ = −0.55, −0.19). In addition, while hangover frequency was significantly increased during L1 (BCa 95% CI_B_ = −0.79, −0.34), hangover severity was significantly lower (BCa 95% CI_B_ = −1.26, −0.55) during L1. No significant differences were seen between the NL1 and BP. During L2, a significant increase in hangover frequency was found (BCa 95% CI_B_ = −0.64, −0.20), whereas hangover severity was significantly lower compared to BP (BCa 95% CI_B_ = −1.05, −0.30). The other drinking outcomes did not significantly differ from BP.

The sample size was too small to further investigate potential sex and age differences, or differences between students and employees.

### 3.2 Smoking

*N* = 59 participants were tobacco smokers. The outcomes on smoking are summarized in Table 3. No significant differences from before the COVID‐19 pandemic were found for the number of smoking days and the number of cigarettes smoked. The sample size was too small to further investigate potential sex and age differences, or differences between students and employees.

**TABLE 3:** Smoking behavior.

Time period	BP	L1	NL1	L2	NL2	Overall
Number of cigarettes smoked per day	3.7 (4.2)	3.8 (4.7)	3.8 (4.0)	4.3 (4.9)	3.2 (3.1)	0.310
Smoking days per week	3.6 (2.8)	3.6 (3.0)	3.7 (2.9)	4.4 (4.2)	4.5 (4.4)	0.082

*Note:* Mean, standard deviation (SD, between brackets), and *p*‐values are shown. The analysis revealed no significant overall main effect (*p* > 0.05). Accordingly, no paired comparisons were conducted.

Abbreviations: BP = before the COVID‐19 pandemic, COVID‐19 = coronavirus disease 2019, L1 = first lockdown period, L2 = second lockdown period, NL1 = first no lockdown period.

## 4 Discussion

The study showed that in comparison to the period before the COVID‐19 pandemic, there were notable differences during the first lockdown in various factors related to alcohol consumption and subsequent hangovers in Germany. There was a significant decrease in weekly alcohol intake, and in the number of days where more than eight alcoholic drinks were consumed. Whereas hangover frequency was significantly increased during L1, hangover severity was significantly reduced. During the first no lockdown period, drinking behaviors returned to BP levels. During the second lockdown period, the decrease in alcohol consumption was much smaller, and not reached statistical significance. Again, hangover frequency was significantly increased whereas hangover severity was significantly reduced. With regards to smoking, no significant differences compared to BP were found.

The results regarding alcohol consumption are in line with our hypothesis and previous studies among students that we conducted in the Netherlands and Argentina (Hendriksen et al. 2021, 2022; Merlo et al. 2021a; Karadayian et al. 2023). However whereas in these countries both hangover frequency and severity were reduced during lockdown periods, in Germany hangover frequency was significantly increased, whereas hangover severity was significantly reduced. While reduced hangover severity is in line with the overall reduction in alcohol consumption and drinking days during the lockdown periods, it is unclear why the increase in hangover frequency was observed in the current study.

Other studies investigating student samples also reported an overall reduction in alcohol consumption during lockdown periods (Reyerson et al. 2021; Evans et al. 2021; Jaffe et al. 2021; Valente et al. 2021; Schmits and Glowacz 2022), in line with general population samples that reported a decrease in the number of weekly consumed alcoholic drinks, number of drinking days, and number of heavy drinking days (White et al. 2020; Merlo et al. 2021b, 2022; Jackson et al. 2022). The closure of venues where alcohol is regularly consumed by young adults (e.g., bars, restaurants, clubs), and the fact that a number of students returned to living with their parents during the COVID‐19 pandemic, may have accounted for the reduction in overall alcohol consumption and smoking. For example, a study among college students found that the percentage of students who decreased their number of alcoholic drinks consumed per occasion during the spring semester of 2020 was much larger among students who relocated from the student residence to their parents' home (49%) compared to students that remained living with other students (21%) (Jaffe et al. 2021).

Of note, 78.6% of our sample reported a reduction or no change in weekly alcohol consumption during the first lockdown period, whereas 21.4% reported an increase in weekly alcohol consumption. These percentages are in line with those observed in the Netherlands (Merlo et al. 2021a) and Argentina (Karadayian et al. 2023). The fact that more than one in five young adults in Germany, increased their alcohol consumption is of concern, as this comprises a substantial number of young adults. It is important to identify the motives for their increased alcohol consumption (e.g., increased loneliness during lockdown periods), in order to develop targeted prevention programs for future pandemics or comparable stressful and isolating situations.

In contrast to the reduction in smoking that was found in Argentina (Karadayian et al. 2023) and the general decline in smoking among German adults since 2003 (Zeiher, Kuntz, and Lange 2017), the current study revealed no significant changes in smoking behavior during the COVID‐19 pandemic. The absence of statistically significant effects on smoking variables in the current study may be due to the small sample size. While the literature showed mixed results for smoking patterns (Guignard et al. 2021; Jackson et al. 2021, 2022; Karadayian et al. 2023), no significant changes in smoking behavior were observed during the COVID‐19 pandemic in the current study. However, it must be noted that in the current study only a small subsample were smokers. Therefore, these findings must be interpreted with caution.

The results should be interpreted taking into account several limitations of the study. Firstly, no a priori sample size was calculated for this study and the final sample size was relatively small. For the overall data on alcohol consumption and smoking behavior, a bootstrapping technique was used to account for this relative small sample size. Unfortunately, the sample size did not allow to conduct reliable statistical comparisons according to sex, age, and occupation status.

Secondly, the survey data were gathered retrospectively through self‐reporting, introducing the potential for inaccuracy and susceptibility to recall bias. While the lockdown period can be considered exceptional, allowing participants to recall its impact relatively well, there remains the possibility that individuals may have exaggerated the effects of the lockdown or idealized the pre‐pandemic period. Thirdly, the sample included participants of both sexes spanning an age range from 18 to 35 years, so that the extent to which this data can be generalized to other age groups or the entire German drinking population remains to be determined in future research.

## 5 Conclusions

During the COVID‐19 lockdown periods in Germany, a significant decrease in alcohol consumption was observed among young adults, along with an increase in hangover frequency and a reduction in hangover severity. Although the evaluated sample was small, no significant changes were found for smoking behavior.

## Author Contributions

Conceptualization, A.M., A.H.K., P.A.H., J.C.V., J.G., G.B., and A.‐K.S.; Data analysis, J.C.V.; Writing original draft, A.M.; Writing–review and editing, A.M., A.H.K., P.A.H., J.C.V., J.G., G.B., and A.‐K.S. All authors have read and agreed to the published version of the manuscript.

## Ethics Statement

The study was conducted in accordance with the Declaration of Helsinki, and approved by the Ethics Committee of the Medical Faculty of the TU Dresden (Approval code: SR‐EK‐8012020, date of approval: 27 September 2021).

## Consent

Informed consent was obtained from all subjects involved in the study.

## Conflicts of Interest

Over the past 3 years, J.C.V. has acted as a consultant for Eisai, KNMP, Med Solutions, Mozand, Red Bull, Sen‐Jam Pharmaceutical, and Toast! J.G. is a part‐time employee of Nutricia Research and has received research grants from Nutricia research foundation, Top Institute Pharma, Top Institute Food and Nutrition, GSK, STW, NWO, Friesland Campina, CCC, Raak‐Pro, and EU. The other authors have no potential conflicts of interest to disclose.

## Data Availability

The dataset is published as attachment to Koyun et al. (2022).
